# Empagliflozin prevents doxorubicin-induced myocardial dysfunction

**DOI:** 10.1186/s12933-020-01040-5

**Published:** 2020-05-15

**Authors:** Jolanda Sabatino, Salvatore De Rosa, Laura Tammè, Claudio Iaconetti, Sabato Sorrentino, Alberto Polimeni, Chiara Mignogna, Andrea Amorosi, Carmen Spaccarotella, Masakazu Yasuda, Ciro Indolfi

**Affiliations:** 1 Division of Cardiology, Department of Medical and Surgical Sciences, Magna Graecia University, Catanzaro, Italy; 2 Cardiovascular Research Center, Magna Graecia University, Catanzaro, Italy; 3 Department of Health Sciences, Magna Graecia University, Catanzaro, Italy; 4 URT CNR of IFC, Magna Graecia University, Catanzaro, Italy

**Keywords:** Heart failure, Cardiotoxicity, Left ventricular function

## Abstract

**Background:**

Empagliflozin showed efficacy in controlling glycaemia, leading to reductions in HbA1c levels, weight loss and blood pressure, compared to standard treatment. Moreover, the EMPA-REG OUTCOME trial demonstrated a 14% reduction of major adverse cardiovascular events (MACE), a 38% reduction in cardiovascular (CV) death and a 35% reduction in the hospitalization rate for heart failure (HF). These beneficial effect on HF were apparently independent from glucose control. However, no mechanistic in vivo studies are available to explain these results, yet. We aimed to determine the effect of empagliflozin on left ventricular (LV) function in a mouse model of doxorubicin-induced cardiomyopathy (DOX-HF).

**Methods:**

Male C57Bl/6 mice were randomly assigned to the following groups: controls (CTRL, n = 7), doxorubicin (DOX, n = 14), DOX plus empagliflozin (DOX + EMPA, n = 14), or DOX plus furosemide (DOX + FURO group, n = 7). DOX was injected intraperitoneally. LV function was evaluated at baseline and after 6 weeks of treatment using high-resolution echocardiography with 2D speckle tracking (Vevo 2100). Histological assessment was obtained using Haematoxylin and Eosin and Masson’s Goldner staining.

**Results:**

A significant decrease in both systolic and diastolic LV function was observed after 6 weeks of treatment with doxorubicin. EF dropped by 32% (p = 0.002), while the LS was reduced by 42% (p < 0.001) and the CS by 50% (p < 0.001). However, LV function was significantly better in the DOX + EMPA group, both in terms of EF (61.30 ± 11% vs. 49.24 ± 8%, p = 0.007), LS (− 17.52 ± 3% vs. − 13.93 ± 5%, p = 0.04) and CS (− 25.75 ± 6% vs. − 15.91 ± 6%, p < 0.001). Those results were not duplicated in the DOX + FURO group. Hearts from the DOX + EMPA group showed a 50% lower degree of myocardial fibrosis, compared to DOX mice (p = 0.03). No significant differences were found between the DOX + FURO and the DOX group (p = 0.103).

**Conclusion:**

Empagliflozin attenuates the cardiotoxic effects exerted by doxorubicin on LV function and remodelling in nondiabetic mice, independently of glycaemic control. These findings support the design of clinical studies to assess their relevance in a clinical setting.

## Background

Empagliflozin (EMPA), a selective inhibitor of the sodium glucose co-transporter 2 (SGLT2), is very effective in controlling glycaemia, leading to a significant reduction in HbA1c levels, blood pressure and body weight, compared to standard optimal treatments [1, 2]. Moreover, the EMPA-REG OUTCOME trial demonstrated a 14% reduction in the primary composite endpoint of major adverse cardiovascular events (MACE), a 38% reduction in cardiovascular (CV) death and a 35% reduction in the hospitalization rate for heart failure (HF) [1–4]. Notwithstanding the beneficial clinical response, the pathophysiological underlying mechanisms are not straightforward [5–7]. Indeed, multiple mechanisms have been suggested to explain the unexpected favourable effect on CV outcomes in heart failure. Hence, it has been speculated that it might be apparently independent from glucose lowering [5–7]. In line with this hypothesis, the DAPA-HF trial found that the incidence of the composite endpoint of worsening of heart failure or death was significantly lower in the dapagliflozin treatment arm, regardless the absence of diabetes [8]. Similarly, treatment with canagliflozin was associated with a 22% reduction of the primary composite endpoint of cardiovascular death or hospitalization for heart failure in the CANVAS study [9]. Most interesting, the benefit seems to be significantly larger in patients with heart failure at baseline. These results point to a protective effect of gliflozin drugs in heart failure, both for type 2 diabetes patients and for nondiabetics, which propelled the development of large clinical trials to evaluate this hypothesis (EMPEROR-Reduced, NCT03057977; EMPEROR-Preserved, NCT03057951; DELIVER, NCT03619213). Meanwhile, the mechanisms underlying these effects remain elusive.

Although, advances in the diagnosis and treatment of cancer have markedly prolonged the survival of cancer patients, the “double edged sword” of novel and traditional cancer chemotherapies is represented by detrimental effects on cardiac function [10]. Beside the consistent efforts for the early identification and treatment of patients who may develop cardiotoxicity [11, 12], the most difficult challenge remains the lack of effective cardioprotective drugs in primary prevention [13, 14]. Currently, there is no molecule with actual cardioprotective effect on anthracycline toxicity, without side effects, reduced sensitivity to cancer treatment or incidence of secondary cancers.

For this reason, we evaluated the impact of empagliflozin on anthracycline-induced cardiotoxicity and studied the underlying molecular mechanisms on adult mammalian hearts. In particular, we spotlighted the involvement of SGLT1 and SGLT2 receptors and the stress signalling pathways, including extracellular signal-regulated kinase (ERK), Janus kinase (JNK), and mitogen-activated protein kinases (MAPKs), in a murine model of cardiotoxicity induced by doxorubicin.

In this context, we aimed to assess the effect of empagliflozin on left ventricular (LV) function in mice with doxorubicin-induced cardiomyopathy (DOX), using high resolution 2-D echocardiography and advanced speckle tracking (STE) analyses.

## Methods

### Animal model

Male 8 weeks old C57Bl/6 mice were housed in plexiglass cages, at 12 h light/dark cycles at 22 ± 2 °C, with free access to food and water. Animal experiments were performed according to European Directive 63/2010/UE and italian DL 26/2014. Chronic myocardial injury was obtained using a standardized model of DOX-induced toxicity, already described in previous studies [15].

### Urine volume and glycosuria measurements by metabolic cages

Ten conscious mice were individually placed in metabolic cages in a soundproof room, randomized to either EMPA (n = 6) or placebo (n = 4). After acclimatization urine volume and water intake were measured for 5 days.

### Standard echocardiographic analyses

In vivo, cardiac function was assessed by transthoracic echocardiography (TTE) using a Vevo 2100 high-resolution imaging system (VisualSonics), with 22–55 hMHz transducer at a frame rate of 233 Hz [11], under light anesthesia [16, 17].

Diastolic- (LVIDd) and systolic-LV diameters (LVIDs) were measured using the M-mode. Fractional shortening (FS = [(LVIDd − LVIDs)/LVIDd] × 100) and ejection fraction (EF = [(EDvol − ESvol)/EDvol] × 100) were calculated.

### Speckle tracking analysis

Acquired B-mode loops were analysed with VevoStrain (VisualSonics) [11].

### Morphological examination and cardiac fibrosis analysis

Hearts were fixed in 10% formalin and embedded in paraffin. Sections were stained with hematoxylin and eosin and Masson’s trichrome staining. Measured were obtained using a Nikon NIS ELEMENTS BRV system.

### Blood pressure measurements

Non-invasive blood pressure measurements were performed in the three study groups at baseline and at the end of the treatment, by means of BP-2000 Blood Pressure Analysis System. Mice were “trained” on the procedure to minimize agitation and pressure values measured as the mean of three measurement after having discarded the first three measurements [18, 19].

### Blood samples analysis

Troponin T (TnT), brain natriuretic peptide (BNP) and glucose were measured on whole blood using the point-of-care Samsung LABGEO PT10.

### Quantitative real-time polymerase chain reaction (PCR)

After RNA extraction, real-time PCR was performed with SYBR-green based detection using an ABI-7500 system (Applied Biosystems) [20]. Results were expressed as fold-change compared to the control.

### Protein extraction and western blot

Proteins were extracted using a lysis buffer containing protease- and phosphatase-inhibitors. Cell lysates were separated by SDS-PAGE and transferred onto nitrocellulose membrane. After treatment with 5% non-fat milk, these were first incubated overnight at 4 °C with primary antibodies (SGLT1, SGLT2, ERK, phospho-ERK), then with secondary antibodies and finally measured by enhanced chemiluminescence. GAPDH and β-actin were used as internal controls.

### Statistical analysis

Continuous data were expressed as mean ± SD. Nonparametric tests were used both for paired and unpaired comparisons [21, 22]. Repeated measures ANOVA was used for all baseline to end-of-study comparisons. A p < 0.05 was considered significant.

## Results

### Baseline characteristics

Fortytwo C57BL/6 mice were assigned to the following groups: CTRL (n = 7), DOX (n = 14), DOX + EMPA (n = 14) and DOX + FURO (n = 7) (Additional file 1: Figure S1). All mice had a structurally and functionally normal heart at baseline (Table 1). Four mice died before the 6-weeks echocardiographic control (primary study endpoint): two from the DOX group, one from the DOX + EMPA group and one from the DOX + FURO group.

**Table 1:** Baseline characteristics

	Baseline mice
Weight, gr	22.21 ± 3.5
Glucose, mg/dl	210.5 ± 50
Systolic pressure, mmHg	122 ± 10
Diastolic pressure, mmHg	81 ± 7
LVEF, %	70.0 ± 7%
LVFS, %	39.6 ± 3%
LVEDd, mm	3.15 ± 0.3
LVESd, mm	1.98 ± 0.6
IVS, mm	0.80 ± 0.003
PW, mm	0.78 ± 0.002
Longitudinal strain (LS), %	− 23.9 ± 4%
Circumferential strain (CS), %	− 30.2 ± 8%
Radial strain (RS), %	43.4 ± 9%
E/A	1.5 ± 0.1
E/Eʹ	41.2 ± 2.3

Values are mean ± SD, or n (%)

*LVEF* left ventricular ejection fraction, *LVFS* left ventricular fractional shortening, *IVS* interventricular septum, *PW* posterior wall

Baseline body weight was 22.2 ± 3.5 g at the beginning of treatment, with no difference between the groups (p = 0.361). There was no difference in body weight at 6 weeks between the groups (p = 0.587) (Table 2).

**Table 2:** Study endpoints at 6 weeks

	Controls	DOX group	DOX + EMPA group	DOX + FURO group
Weight, g	23.18 ± 1.8	21.45 ± 2.7	22.5 ± 2.1	21.8 ± 2.4
Glucose, mg/dl	200.6 ± 40	166.6 ± 72	205.5 ± 39	170.5 ± 50
Systolic pressure, mmHg	121 ± 15	102 ± 37	119 ± 20*	105 ± 33
Diastolic pressure, mmHg	76 ± 11	41 ± 10	65 ± 28*	60 ± 24*
LVEF, %	68.7 ± 5%	49.24 ± 8	61.30 ± 11*	55.79 ± 11
LVFS, %	39.4 ± 3%	24.70 ± 5	33.08 ± 8*	28.62 ± 8
LVEDd, mm	3.11 ± 0.1	3.98 ± 0.4	3.89 ± 0.3	3.80 ± 0.7
LVESd, mm	1.93 ± 0.5	2.98 ± 0.4	2.67 ± 0.5	2.73 ± 0.5
IVS, mm	0.81 ± 0.001	0.69 ± 0.004	0.77 ± 0.001*	0.77 ± 0.005*
PW, mm	0.78 ± 0.001	0.71 ± 0.005	0.76 ± 0.013	0.76 ± 0.014
Longitudinal strain (LS),%	− 24.1 ± 4%	− 13.93 ± 5	− 17.52 ± 3*	− 16.1 ± 3.5
Circumferential strain (CS), %	− 29.9 ± 8%	− 15.91 ± 6	− 25.75 ± 6*	− 15.8 ± 3.3
Radial strain (RS), %	46.0 ± 7%	26.7 ± 13	24.0 ± 5	26.1 ± 4
E/A	1.6 ± 0.1	1.7 ± 0.2	1.6 ± 0.2	1.5 ± 0.1
E/Eʹ	41.1 ± 2.2	77.3 ± 3.5	61.1 ± 5.7*	59.4 ± 4*

Values are mean ± SD, or n (%)

*LVEF* left ventricular ejection fraction, *LVFS* left ventricular fractional shortening, *IVS* interventricular septum, *PW* posterior wall

*p < 0.05 compared to DOX

### Blood pressure

C57BL/6 mice had normal blood pressure (BP) at baseline (122 ± 10/81 ± 7 mmHg), with no difference between the groups (p = 0.36). At 6 weeks, systolic BP (sysBP) (p < 0.001) and diastolic BP (diaBP) (p < 0.001) were lower in DOX-treated mice compared to controls (Additional file 1: Figure S2). This drop in blood pressure was attenuated in the DOX + EMPA group, where both sysBP (119 ± 20 vs. 102 ± 37; p = 0.023) and diaBP (65 ± 28 vs. 41 ± 10; p < 0.001) were significantly higher compared to DOX mice (Additional file 1: Figure S2). No significant difference was observed for sysBP between DOX + FURO and DOX mice (105 ± 33 vs. 102 ± 37; p = 0.705) (Additional file 1: Figure S2A). DiaBP in the DOX + FURO group was significantly higher than in DOX mice (60 ± 24 vs. 41 ± 10; p = 0.001), although this difference was numerically smaller compared to DOX + EMPA animals (Additional file 1: Figure S2B).

### Glycemia and glycosuria

Mean basal blood glucose level was 210.5 ± 50 mg/dl, with no difference between the groups (p = 0.28). Similarly, no significant difference was observed between the groups at the end of treatment (p = 0.37).

In a parallel experiment involving ten mice in metabolic cages, we did not find significant differences in glycosuria or urine volume between EMPA mice (n = 6) and controls (n = 4) (p = 0.31).

### Effects on systolic LV function

At the sixth week of treatment, mice treated with DOX presented a significant reduction of multiple LV function parameters compared to baseline. In particular, we found a 32% reduction in EF (p = 0.002), a 36% reduction in FS (p = 0.002), a 29% increase in LVEDd (p = 0.002) a 50% increase in LVESd (p = 0.002).

**Fig. 1:**
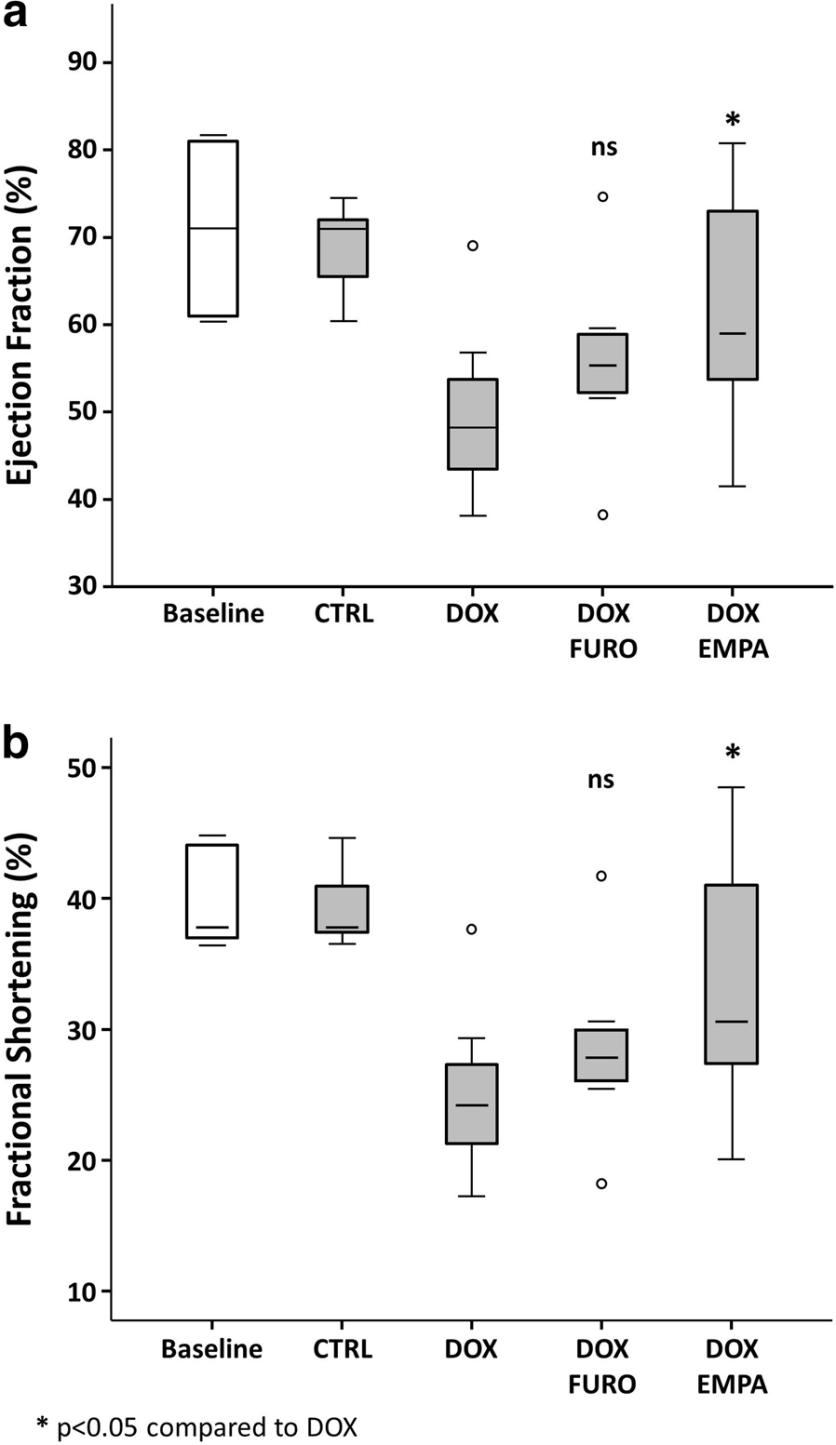
LV systolic function. Mice treated with DOX presented a significant reduction of LV function parameters compared to baseline. On the other hand, mice in the DOX + EMPA group had a significantly better LV function compared to the DOX group

At 6 weeks, mice in the DOX + EMPA group had a significantly better LV function compared to the DOX group, both in terms of EF (61.30 ± 11% vs. 49.24 ± 8%, p = 0.007) and FS (33.08 ± 8% vs.. 24.70 ± 5%, p = 0.008) (Fig. 1).

No significant differences were observed in EF and FS between the DOX + FURO and the DOX groups, as measured by EF (55.79 ± 11% vs. 49.24 ± 8%, p = 0.188) or FS (28.62 ± 8% vs. 24.70 ± 5%, p = 0.221).

**Fig. 2:**
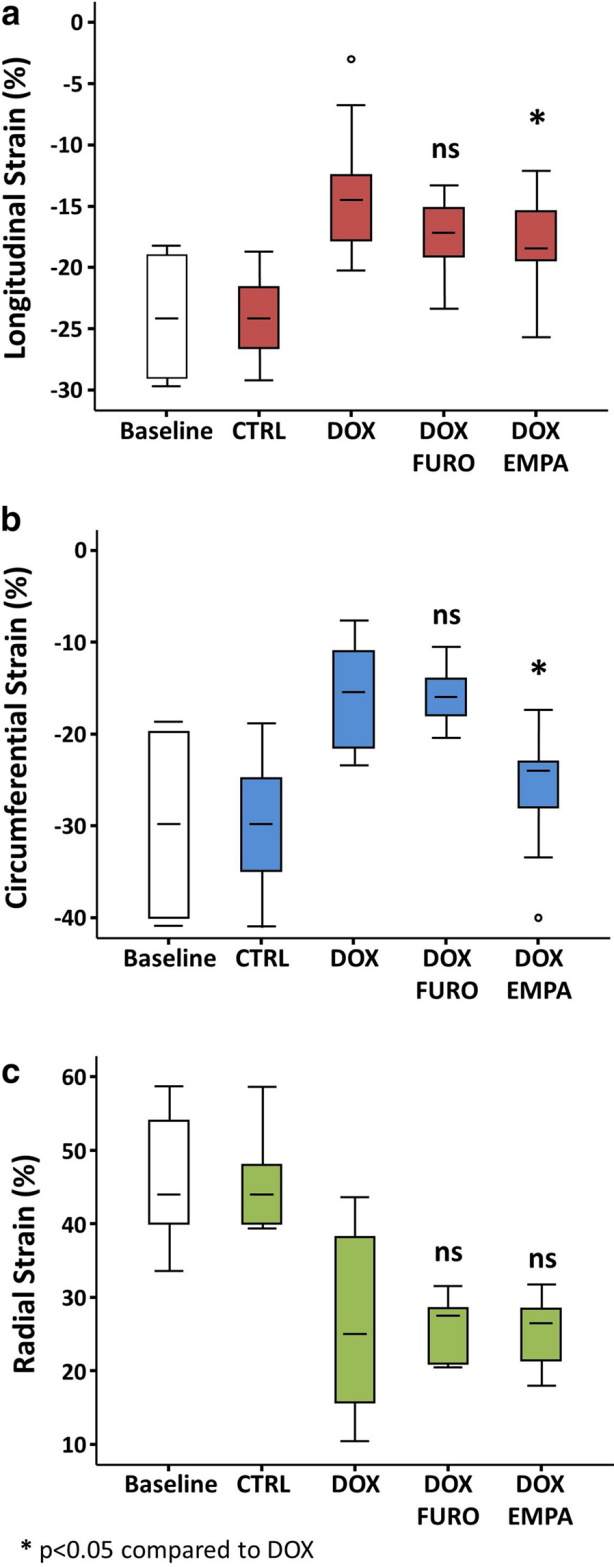
Speckle tracking analysis. LV strain parameters were severely impaired in the DOX group compared to baseline. Moreover, DOX + EMPA mice had significantly better longitudinal and circumferential strains values compared to the DOX group

LV strain parameters were significantly impaired in the DOX group compared to baseline (Fig. 2). In details, we found a significant worsening of longitudinal strain (LS) from − 23.9 ± 4% to − 13.93 ± 5% (p < 0.001) of circumferential (CS) from − 30.2 ± 8% to − 15.91 ± 6% (p < 0.001) and of radial strain (RS) from 43.4 ± 9% to 26.7 ± 13% (p < 0.001). At the sixth week of treatment, DOX + EMPA mice had significantly better LS (− 17.52 ± 3% vs. − 13.93 ± 5%, p = 0.04) (Fig. 2a) and CS values (− 25.75 ± 6% vs. − 15.91 ± 6%, p < 0.001) (Fig. 2b) compared to DOX mice. No significant difference in RS (Fig. 2c). DOX + FURO mice presented no significant difference in mean LS, CS, RS compared to DOX (Fig. 2).

### Effects on diastolic LV function

**Fig. 3:**
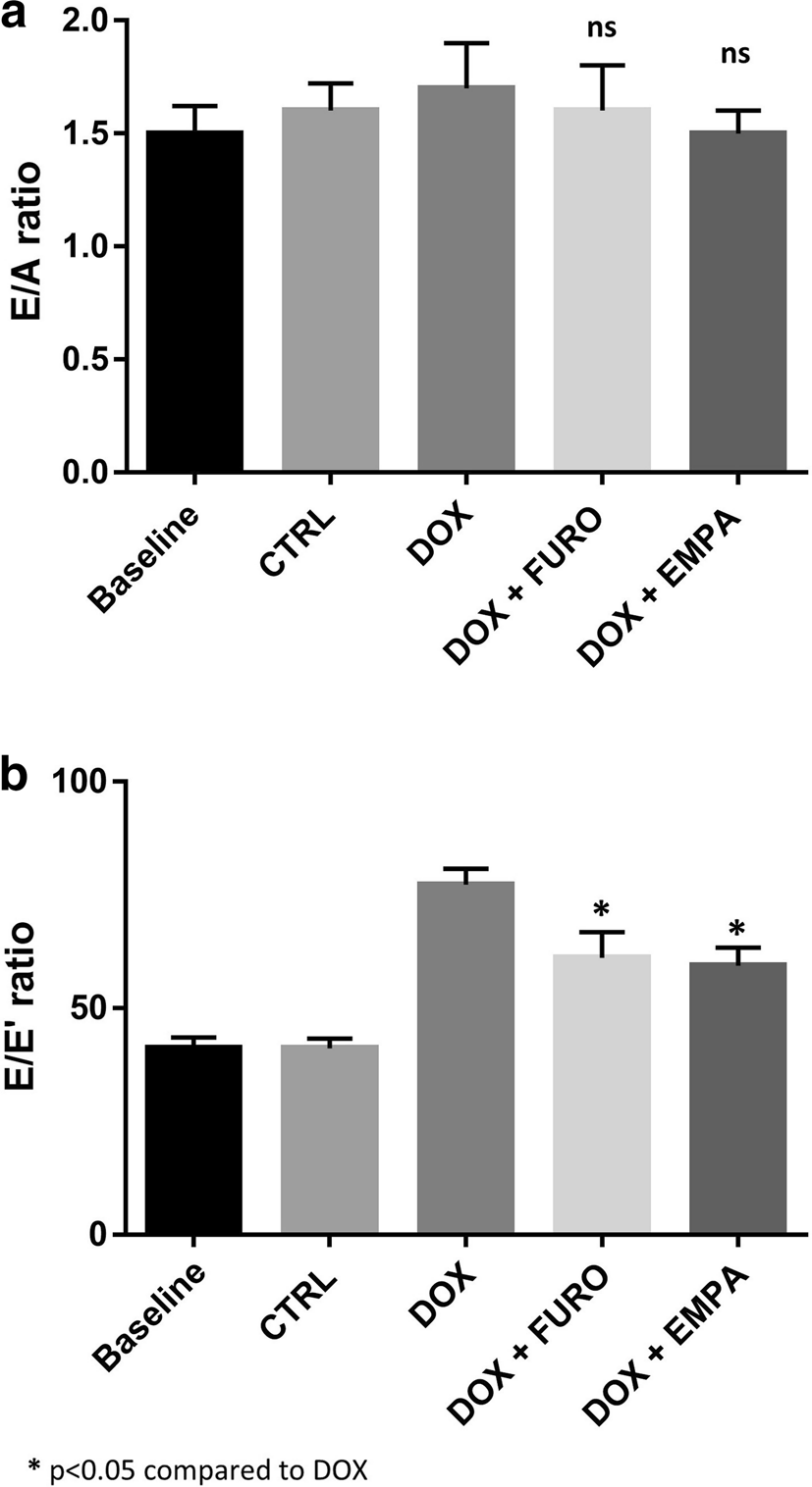
LV diastolic function. **a** Shows results of E/A Ratio across the groups. **b** Shows results of E/Eʹ ratio

No significant differences were observed in the ratio of the early (E) to late (A) ventricular filling velocities (E/A) between the experimental groups (Table 2, Fig. 3). On the other hand, treatment with doxorubicin was associated with a significant reduction of the early mitral inflow velocity to mitral annular early diastolic velocity (E/Eʹ) ratio (p < 0.001). Both DOX + EMPA (p < 0.001) and DOX + FURO (p < 0.001) mice had a lower E/Eʹ ratio compared to the DOX group (Table 2, Fig. 3).

### Effects on myocardial fibrosis and LV remodelling

**Fig. 4:**
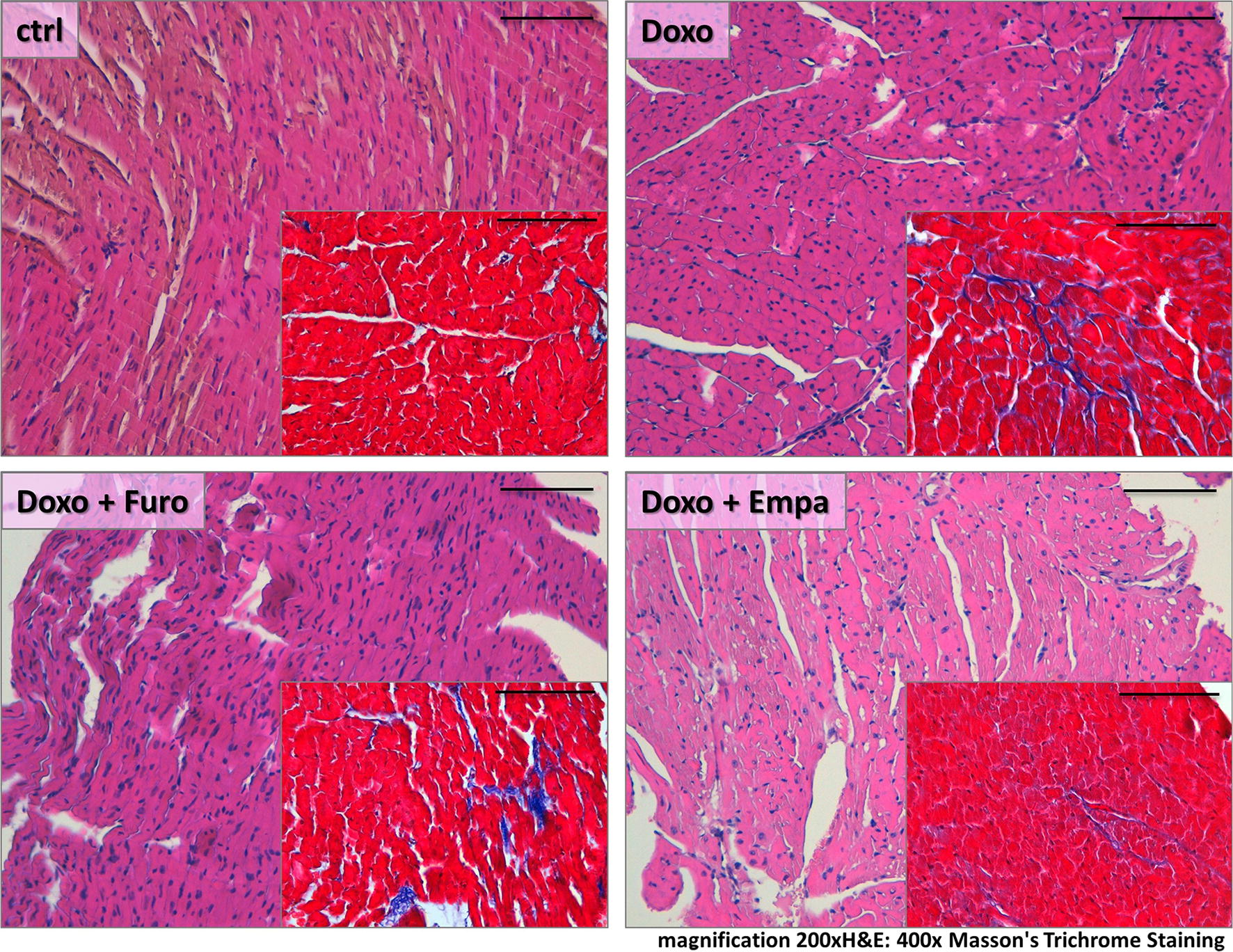
Histological sections. The figure shows the effects on myocardial fibrosis and remodelling in the different study groups compared to controls (CTRL)

Mice were sacrificed after 7 weeks of treatment with doxorubicin. Masson’s trichrome staining revealed that hearts from DOX + EMPA mice (insert of lower right panel, Fig. 4) had a 50% lower degree of myocardial fibrosis (p = 0.03) compared to DOX mice (insert of upper right panel, Fig. 4). On the contrary, treatment with furosemide (DOX + FURO) (insert of lower left panel, Fig. 4) was not associated to a significant change in myocardial fibrosis (p = 0.103).

The characteristic attenuation of fibrillar bands observed after treatment with doxorubicin (92% in DOX mice) (upper mid panel, Additional file 1: Figure S3) was prevented by 2.3-fold to 40% (p = 0.010) by the concomitant treatment with empagliflozin (DOX + EMPA), while furosemide had no effect (75% in DOX + FURO mice; p = 0.880).

Severe disarray of myocardial fibers was more frequent in DOX (67%) compared to DOX + EMPA mice (30%; p = 0.087). No significant difference was observed between FURO and DOX (p = 0.800).

Wavy myocardial fibers were significantly reduced in DOX + EMPA (40%), compared to DOX (100%; p = 0.002). No significant difference was observed between DOX + FURO (75%) compared to DOX (p = 0.054).

Vacuolization of myocytes was observed in 100% of fields from DOX mice, in 60% in DOX + EMPA (p = 0.015) and 75% of DOX + FURO (p = 0.054).

### Troponin T and BNP levels in C57BL/6 mice

Mean blood troponin T (TnT) in C57BL/6 mice was 0.19 ± 0.05 ng/ml at baseline. A significant increase in TnT values was observed at the end of the study in the DOX group (TnT = 0.70 ± 0.32; p = 0.028), but not the the DOX + EMPA (TnT = 0.22 ± 0.18; p = 0.317), nor in the DOX + FURO (TnT = 0.46 ± 0.26; p = 0.109) groups.

Baseline BNP values were below the detection limit of the qualitative assay used (< 30 pg/ml) at baseline. BNP values were increased above the positivity threshold of the assay used (> 150 pg/ml) in all three study groups.

### Expression of SGLT2 in mice heart

**Fig. 5:**
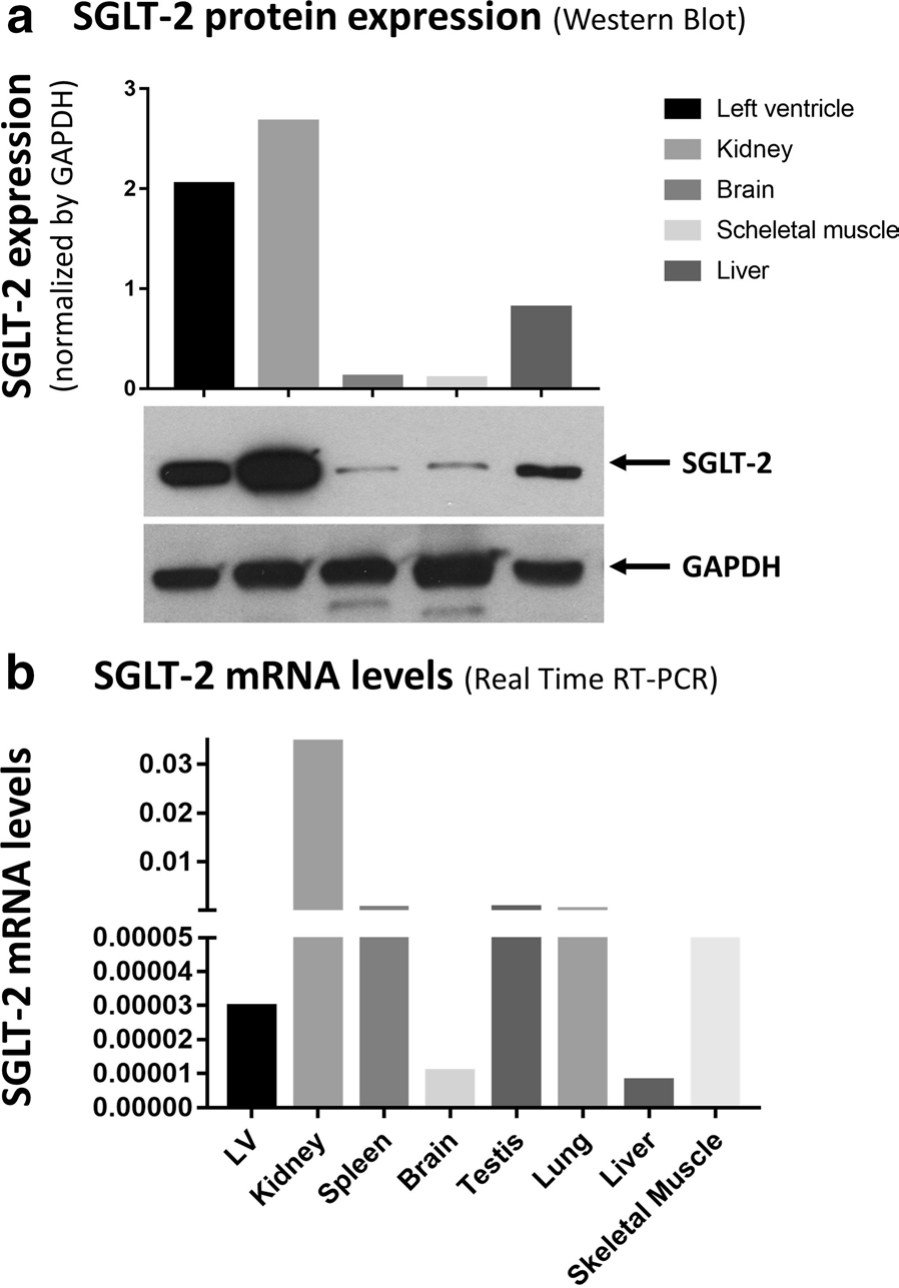
SGLT2 in mice heart. **a** Shows the expression of SGLT2 at western blot (the insert report a cropped picture of the blotting plate). **b** Reports results of RT-PCR analysis

Expression of SGLT2 at western blot showed a substantial variation across different organs and tissues, with the highest expression levels in kidneys followed by the left myocardium (Fig. 5a). RT-PCR confirmed detectable levels of the SGLT2 mRNA in LV myocardium from study animals (Fig. 5b). Similar results were observed for SGLT1 (Additional file 1: Figure S4).

Immunohistochemistry confirmed the expression of SGLT2 in mice hearts (Additional file 1: Figure S5). SGLT2 expression showed a 75% reduction in DOX mice, compared to baseline (p < 0.001). This effect was significantly attenuated in EMPA + DOX mice (20% reduction compared to ctrl) (p = 0.263).

### Signalling pathways modulated by empagliflozin treatment

**Fig. 6:**
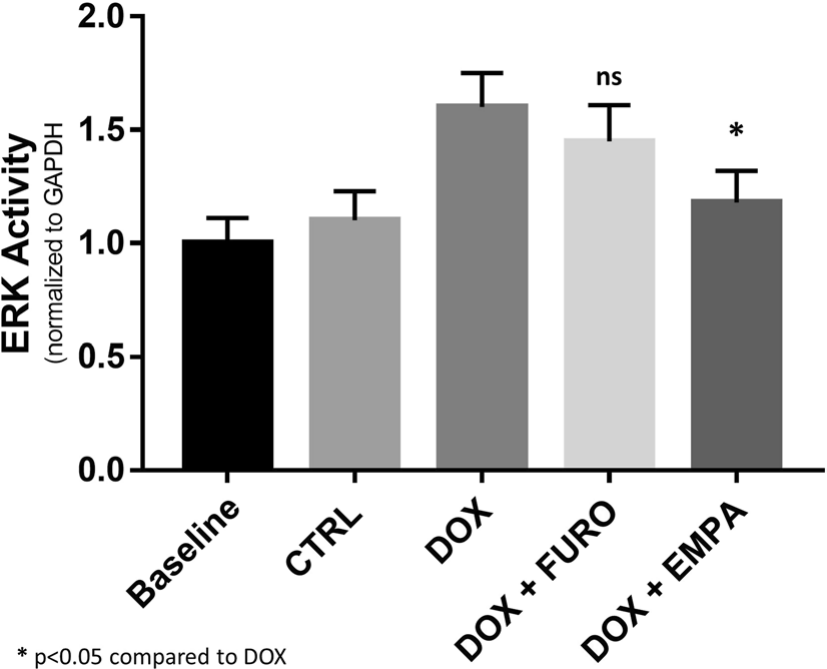
LV ERK activity. The bar graph reports mean ERK Activity across all study groups

As ERK is a known pharmacological target of empagliflozin, we measured ERK activity in myocardial tissue samples obtained from all mice. ERK activity was significantly increased in DOX mice compared to controls (p = 0.018). Interestingly, treatment with empagliflozin (EMPA + DOX) was associated with a 36%-lower level of ERK activity compared with doxorubicin alone (DOX) (p = 0.038). On the contrary, the additional treatment with furosemide (FURO + DOX) had no significant impact on ERK activity compared to doxorubicin treatment alone (DOX) (12% reduction in ERK activity; p = 0.177) (Fig. 6).

## Discussion

Our results demonstrate that the treatment with empagliflozin, a SGLT2 inhibitor, prevents the reduction in cardiac systolic function induced by a cardiotoxic anthracycline in a model of non-diabetic mice. The protective impact of empagliflozin on systolic function was also associated with better systolic and diastolic blood pressures in mice treated with empagliflozin compared to those treated with doxorubicin alone. Finally, histological examination showed a lower degree of myocardial fibrosis and fibres disarray in mice treated with empagliflozin.

### Effects of empagliflozin on LV function

The improvement in LV systolic function recorded in our study, not only by 2-D high resolution echocardiography but also by STE, provides a potential explanation for the unexpected favorable cardiovascular outcomes of SGLT2 inhibitors both in T2DM and in non-diabetics in recent clinical trials [4, 8, 9]. Our results, confirm and extend recent evidence in different animal models and experimental conditions. In fact, SGLT2 were associated to reverse LV remodeling by attenuating sodium and calcium dysregulation [23, 24], mitigation of atrial remodeling [25], attenuation of negative effects on electric LV remodeling [26], normalization of LV mitochondrial function [27] and reduced mortality in diabetic rats [23]. More recently, empagliflozin was shown to improve post-MI cardiac function in non-diabetic rats [28] and to improve LV hemodynamics in hypertensive rats [29]. Very recently, empagliflozin was shown to improve cardiac contractility alongside microvascular function in pre-diabetic or early-stage diabetic mice [30].

### Effects of empagliflozin on cardiac fibrosis

Previous animal studies demonstrated that empagliflozin improves cardiac diastolic function in a female murine model of T2DM, db/db mice [16]. Similar results were independently obtained on diabetic mice through the attenuation of oxidative stress [31]. Accordingly, the improvement in diastolic function was associated with improved glycemia, but also with a positive LV remodeling, such as reduction in interstitial myocardial fibrosis and attenuation of cardiomyocyte hypertrophy [16]. In line with the above reported studies, we found reduced myocardial fibrosis also in mice with DOX-induced cardiomyopathy that received a concomitant treatment with empagliflozin. However, our results extend those observations to demonstrate a protective effect in non-diabetic individuals, opening promising new perspectives for the prevention and the treatment of anthracycline-induced cardiotoxicity in patients treated with antitumoral chemotherapy, such as in breast cancer.

### Pathophysiological mechanisms

The pathophysiological mechanisms underlying the remarkable cardiac effects of empagliflozin in the EMPA-REG OUTCOME trial are still unresolved. The SGLTs are a family of proteins able to translocate glucose in different tissues [32]. Both SGLT2 and SGLT1 co-transport Na+ together with glucose. While SGLT1 is expressed in small intestine, lungs, kidneys, liver and cardiac myocytes, SGLT2 has been primarily found in the kidney, but it is also expressed by pancreatic alpha cells. We demonstrated that SGLT2 is expressed in murine cardiac myocytes of C57BL/6 mice. Notwithstanding the previous report by Yoshii et al., that did not find transcription of the SGLT2 gene in mice hearts [33], we were able to demonstrate SGLT2 expression in C57BL/6 mice hearts using 3 different methodologies: transcription as shown by RT-PCR (Fig. 5b); protein expression, using western blotting (Fig. 5a); and immunohistochemistry (Additional file 1: Figure S5). This apparent discrepancy of results might be related to some differences between the work by Yoshii and colleagues and ours [33]. First, they only performed PCR experiments (n = 3), whereas we found consistent results using two different techniques. It should be noted that protein expression levels we found were higher than one might have expected from SGLT2 transcription levels, suggesting that post-transcriptional regulation might be particularly relevant to SGLT2 expression in this model. In this regard, it should be mentioned that SGLT2 is target of multiple microRNAs, a class of post-transcriptional regulators of gene expression (Additional file 1: Table S1). In particular, the highly conserved miR-296-5p, recently found to be involved in the modulation of cardiac hypertrophy [34] and myocardial fibrosis [35] was able to promote healing of diabetic wounds, directly targeting SGLT2 [36]. In addition, SGLT2 translation is also influenced by glucose level [37]. Our findings of SGLT2 expression in mice are in line with the recent demonstration of expression SGLT2 in both mouse [38] and human heart tissue [39]. The same authors also report that high glucose levels were associated with a significant increase in expression levels of both of SGLT1 (7.1 folds; p < 0.01) and SGLT2 (7.5-folds; p < 0.01) and that treatment with empagliflozin was able to restore physiological SGLT1 and SGLT2 expression levels, opening the way for potential relevance of our finding in humans [39].

The expression of functional SGLT1 in human hearts was already demonstrated by von Lewinsky et al. [40], that reported that SGLT1 contributes to the positive inotropic effect exerted by insulin in patients with end-stage HF. Hence, our direct targeting of cardiac SGLT2 receptors is a further potential mechanism.

Therefore, our hypothesis is that SGLT2 inhibition may affect the tissue and cellular Na+ homeostasis that is responsible for the excitation–contraction coupling and the mitochondrial redox regulation in cardiomyocytes. In the 2000s Despa et al. [41] observed that [Na+]i is increased in failing cardiac myocytes, as a consequence of increased Na+ influx via the late Na+ current, increased activity of the sarcolemmal Na+/H+ exchanger (NHE), and a reduction in Na+/K+ ATPase activity. Moreover, SGLT1 expression in the heart was found to be upregulated both in animal models of type 2 diabetes and in patients with diabetic cardiomyopathy, and its activity contributes to the increase in [Na+]i.

Moreover, Baartscheer et al. recently reported that EMPA reduced [Na+]i and [Ca2+]c in isolated ventricular myocytes independently of the presence of glucose [42]. In line with this pathophysiological hypothesis, the beneficial effect exerted by EMPA in our experimental model was independent of any significant impact on glycemia, glycosuria or diuresis ad demonstrated by the experiments performed using metabolic cages.

EMPA is reported to reduce glycemia by increasing glycosuria and natriuresis in diabetic humans [43]. However, in line with human findings we did not observe significantly lower glycemia in EMPA-treated non-diabetic mice. Furthermore, in line with the latter findings we did not observe an increase in glycosuria or diuresis compared to mice not receiving EMPA, thus excluding that the effect of EMPA on LV structure and function could have been mediated by changes in blood volume or loading conditions. Hence, cardiac functional and structural improvements in EMPA-treated non-diabetic mice occurred independently of glycemia, glycosuria or diuresis.

In light of these considerations, it is tempting to speculate that our positive results on LV function in non-diabetic mice may be—at least in part—related to direct effects of EMPA on cardiac ion homeostasis. By decreasing [Na+]i and restoring mitochondrial Ca2+ handling, EMPA may ameliorate mitochondrial energetic mismatch and production of ROS, thus interrupting the vicious circle which underlies Na+ overload and oxidative stress in cardiomyocytes.

### Study limitations

We analysed blood levels of Troponin, BNP and glucose under basal conditions and 6 weeks later. Since in mice they are obtained from the retro-orbital vein, the blood samples volumes were not quantitatively adequate to be analysed by standard ELISA assays. To overcome this limitation we used a point-of-care analyzer (Samsung LABGEO PT10, Thermo Fischer Scientific, Henningsdorf, Germany). However, we could not measure very high or low levels of BNP and this is due to the technical limitations of this method. In fact, we found that baseline BNP values were < 30 pg/ml at baseline; on the other hand, a significant increase in BNP blood levels was observed at the end of doxorubicin treatment (> 150 pg/ml in all the study groups). Pressure measurement was performed using the tail-cuff method in this study, to avoid the surgical procedure to implant telemetric devices. Nevertheless, this method presents some limitations in mice, which are mostly related to the agitation caused by the restraint. It should be noticed that blood pressure was not a key outcome in this study. However, some measures were adopted to minimize variability in measurements: mice were “trained” on the procedure, pressure values measured as the mean of three measurement after having discarded the first three measurements.

### Clinical perspective

Our results might have an impact on the development of a novel treatment for antineoplastic therapy-induces cardiotoxicity, a relevant unmet need in medicine. In fact, despite the increasingly target selectivity of antineoplastic treatments, the risk of serious cardiotoxic effects treatment is still a clinical issue. In particular, no effective cardioprotective treatment is available, yet, to contrast anthracycline- and/or trastuzumab-induced toxicity [44]. Several therapeutic approaches have been attempted, including beta-blockers and angiotensin antagonists [45], spironolactone [46] and statins [47]. However, none of these studies could reliably demonstrate a protective effect, leaving cardio-oncologists with a relevant unsolved issue. In fact, up to one quarter of women with breast cancer are ErbB^2^ positive and are therefore eligible for treatment with anthracycline–trastuzumab, which is associated to a higher rate (28%) of heart failure [48]. Unfortunately, results from recent primary prevention studies using single- or multiple-drug approach were also disappointing [49–51].

## Conclusion

Empagliflozin, independently from the glycaemic lowering, prevents the cardiotoxic side effects exerted by doxorubicin on LV function and remodelling in a model of doxorubicin-induced cardiotoxicity in non-diabetic mice. These findings strongly support the design of clinical studies to assess their relevance in a clinical setting.

## Supplementary information

**Additional file 1.Extended methods.** Extended version of study methodology, including all details that the limited word count available did not allow to keep in the main manuscript. **Table S1. MicroRNAs targeting SGLT2.** List of microRNAs potentially targeting SGLT2 within the highest context score percentiles. The upper row shows the microRNA targeting a conserved site of SGLT2, while the remaining microRNAs target poorly conserved sites. **Figure S1. Study timeline.** The figure depicts the study timeline with the breakdown of mice into three groups. **Figure S2. Systemic blood pressure.** At the sixth week, both systolic (panel A) and diastolic blood pressure (panel B) was significantly lower in the DOX group compared to DOX+EMPA. **Figure S3. Histological examples of doxorubicin myocardial injuries. **The figure shows the characteristic attenuation of fibrillar bands observed after treatment with doxorubicin, in some morphological variants (lower left and mid panels and upper mid panel). Masson’s trichrome staining highlights the degree of fibrosis in hearts from control group (upper right panel) and hearts from the DOX group (lower right panel). **Figure S4. Expression of SGLT-1 in mice hearts.** Results RT-PCR showing the expression levels of SGLT-1 in different mouse hearts. **Figure S5. Expression of SGLT-2 in mice hearts.** Immunohistochemistry shows the expression of SGLT-2 in mice hearts. SGLT-2 expression showed a 75% reduction in DOX mice (upper right panel), compared to baseline. This effect was significantly attenuated in EMPA+DOX mice (lower right panel).

## Data Availability

Additional data will be made available to all readers upon request per email.

## Abbreviations

MACE: Major adverse cardiovascular events
CV: Cardiovascular
HF: Heart failure
DOX: Doxorubicin
DOX-HF: Doxorubicin-induced cardiomyopathy
DOX + EMPA: Doxorubicin plus empagliflozin
DOX + FURO: Doxorubicin plus furosemide
STE: Speckle tracking echocardiography
LV: Left ventricular
EF: Ejection fraction
LS: Longitudinal strain
CS: Circumferential strain
EMPA: Empagliflozin
SGLT2: Selective inhibitor of the sodium glucose co-transporter 2
SGLT1: Selective inhibitor of the sodium glucose co-transporter 1
ERK: Extracellular signal-regulated kinase
JAK: Janus kinase
MAPK: Mitogen-activated protein kinases
TTE: Transthoracic echocardiography
LVIDd: Left ventricular internal dimension in diastole
LVIDs: Left ventricular internal dimension in systole
FS: Fractional shortening
TnT: Troponin T
BNP: Brain natriuretic peptide
PCR: Polymerase chain reaction
SD: Standard deviations
sysBP: Systolic blood pressure
diaBP: Diastolic blood pressure
RS: Radial strain
NHE: Na+/H+ exchanger
ROS: Reactive oxygen species

## References

[1] Seshasai SR, Kaptoge S, Thompson A, Di Angelantonio E, Gao P, Sarwar N, Whincup PH, Mukamal KJ, Gillum RF, Holme I, Njølstad I, Fletcher A, Nilsson P, Lewington S, Collins R, Gudnason V, Thompson SG, Sattar N, Selvin E, Hu FB, Danesh J, Emerging Risk Factors Collaboration (2011). Diabetes mellitus, fasting glucose, and risk of cause-specific death. N Engl J Med.

[2] Zinman B, Wanner C, Lachin JM, Fitchett D, Bluhmki E, Hantel S, Mattheus M, Devins T, Johansen OE, Woerle HJ, Broedl UC, Inzucchi SE, EMPA-REG OUTCOME Investigators (2015). Empagliflozin, cardiovascular outcomes, and mortality in type 2 diabetes. N Engl J Med.

[3] Marx N, McGuire DK (2016). Sodium-glucose cotransporter-2 inhibition for the reduction of cardiovascular events in high-risk patients with diabetes mellitus. Eur Heart J.

[4] Fitchett D, Zinman B, Wanner C, Lachin JM, Hantel S, Salsali A, Johansen OE, Woerle HJ, Broedl UC, Inzucchi SE, EMPA-REG OUTCOME trial investigators (2016). Heart failure outcomes with empagliflozin in patients with type 2 diabetes at high cardiovascular risk: results of the EMPA-REG OUTCOME trial. Eur Heart J.

[5] Packer M, Anker SD, Butler J, Filippatos G, Zannad F (2017). Effects of sodium-glucose cotransporter 2 inhibitors for the treatment of patients with heart failure: proposal of a novel mechanism of action. JAMA Cardiol.

[6] Rajasekeran H, Lytvyn Y, Cherney DZ (2016). Sodium-glucose cotransporter 2 inhibition and cardiovascular risk reduction in patients with type 2 diabetes: the emerging role of natriuresis. Kidney Int.

[7] Heerspink HJ, Perkins BA, Fitchett DH, Husain M, Cherney DZ (2016). Sodium glucose cotransporter 2 inhibitors in the treatment of diabetes mellitus: cardiovascular and kidney effects, potential mechanisms, and clinical applications. Circulation.

[8] McMurray JVC, Solomon SD, Inzucchi SE, Køber L, Kosiborod MN, Martinez FA, Ponikowski P, Sabatine MS, Anand IS, Bělohlávek J, Böhm M, Chiang CE, Chopra VK, de Boer RA, Desai AS, Diez M, Drozdz J, Dukát A, Ge J, Howlett JG, Katova T, Kitakaze M, Ljungman CEA, Merkely B, Nicolau JC, O’Meara E, Petrie MC, Vinh PN, Schou M, Tereshchenko S, Verma S, Held C, DeMets DL, Docherty KF, Jhund PS, Bengtsson O, Sjöstrand M, Langkilde AM, DAPA-HF Trial Committees and Investigators (2019). Dapagliflozin in patients with heart failure and reduced ejection fraction. N Engl J Med.

[9] Rådholm K, Figtree G, Perkovic V, Solomon SD, Mahaffey KW, de Zeeuw D, Fulcher G, Barrett TD, Shaw W, Desai M, Matthews DR, Neal B (2018). Canagliflozin and heart failure in type 2 diabetes mellitus: results from the CANVAS program (Canagliflozin Cardiovascular Assessment Study). Circulation.

[10] Brana I, Tabernero J (2010). Cardiotoxicity. Ann Oncol.

[11] Coppola C, Riccio G, Barbieri A, Monti MG, Piscopo G, Rea D, Arra C, Maurea C, De Lorenzo C, Maurea N (2016). Antineoplastic-related cardiotoxicity, morphofunctional aspects in a murine model: contribution of the new tool 2D-speckle tracking. Onco Targets Ther.

[12] Oliveira MS, Melo MB, Carvalho JL, Melo IM, Lavor MS, Gomes DA, de Goes AM, Melo MM (2013). Doxorubicin cardiotoxicity and cardiac function improvement after stem cell therapy diagnosed by strain echocardiography. J Cancer Sci Ther.

[13] Liu J, Banchs J, Mousavi N, Plana JC, Scherrer-Crosbie M, Thavendiranathan P, Barac A (2018). Contemporary role of echocardiography for clinical decision making in patients during and after cancer therapy. JACC Cardiovasc Imaging.

[14] Schneeweiss A, Chia S, Hickish T, Harvey V, Eniu A, Hegg R, Tausch C, Seo JH, Tsai YF, Ratnayake J, McNally V, Ross G, Cortés J (2013). Pertuzumab plus trastuzumab in combination with standard neoadjuvant anthracycline-containing and anthracycline-free chemotherapy regimens in patients with HER2-positive early breast cancer: a randomized phase II cardiac safety study (TRYPHAENA). Ann Oncol.

[15] Hullin R, Métrich M, Sarre A, Basquin D, Maillard M, Regamey J, Martin D (2018). Diverging effects of enalapril or eplerenone in primary prevention against doxorubicin-induced cardiotoxicity. Cardiovasc Res.

[16] Habibi J, Aroor AR, Sowers JR, Jia G, Hayden MR, Garro M, Barron B, Mayoux E, Rector RS, Whaley-Connell A, DeMarco VG (2017). Sodium glucose transporter 2 (SGLT2) inhibition with empagliflozin improves cardiac diastolic function in a female rodent model of diabetes. Cardiovasc Diabetol.

[17] Tsai TH, Lin CJ, Hang CL, Chen WY (2019). Calcitriol attenuates doxorubicin-induced cardiac dysfunction and inhibits endothelial-to-mesenchymal transition in mice. Cells.

[18] Zhao X, Ho D, Gao S, Hong C, Vatner DE, Vatner SF (2011). Arterial pressure monitoring in mice. Curr Protoc Mouse Biol..

[19] Wang Y, Thatcher SE, Cassis LA (2017). Measuring blood pressure using a noninvasive tail cuff method in mice. Methods Mol Biol.

[20] De Rosa S, Eposito F, Carella C, Strangio A, Ammirati G, Sabatino J, Abbate FG, Iaconetti C, Liguori V, Pergola V, Polimeni A, Coletta S, Gareri C, Trimarco B, Stabile G, Curcio A, Indolfi C, Rapacciuolo A (2018). Transcoronary concentration gradients of circulating microRNAs in heart failure. Eur J Heart Fail.

[21] Li H, Johnson T (2014). Wilcoxon’s signed-rank statistic: what null hypothesis and why it matters. Pharm Stat.

[22] Vickers AJ (2005). Parametric versus non-parametric statistics in the analysis of randomized trials with non-normally distributed data. BMC Med Res Methodol.

[23] Lee TI, Chen YC, Lin YK, Chung CC, Lu YY, Kao YH, Chen YJ (2019). Empagliflozin attenuates myocardial sodium and calcium dysregulation and reverses cardiac remodeling in streptozotocin-induced diabetic rats. Int J Mol Sci.

[24] Zhou Y, Wu W (2017). The sodium-glucose co-transporter 2 inhibitor, empagliflozin, protects against diabetic cardiomyopathy by inhibition of the endoplasmic reticulum stress pathway. Cell Physiol Biochem.

[25] Shao Q, Meng L, Lee S, Tse G, Gong M, Zhang Z, Zhao J, Zhao Y, Li G, Liu T (2019). Empagliflozin, a sodium glucose co-transporter-2 inhibitor, alleviates atrial remodeling and improves mitochondrial function in high-fat diet/streptozotocin-induced diabetic rats. Cardiovasc Diabetol.

[26] Durak A, Olgar Y, Degirmenci S, Akkus E, Tuncay E, Turan B (2018). A SGLT2 inhibitor dapagliflozin suppresses prolonged ventricular-repolarization through augmentation of mitochondrial function in insulin-resistant metabolic syndrome rats. Cardiovasc Diabetol.

[27] Mizuno M, Kuno A, Yano T, Miki T, Oshima H, Sato T, Nakata K, Kimura Y, Tanno M, Miura T (2018). Empagliflozin, an SGLT2 inhibitor, reduced the mortality rate after acute myocardial infarction with modification of cardiac metabolomes and antioxidants in diabetic rats. Physiol Rep.

[28] Yurista SR, Silljé HHW, Oberdorf-Maass SU, Schouten EM, Pavez Giani MG, Hillebrands JL, van Goor H, van Veldhuisen DJ, de Boer RA, Westenbrink BD (2019). Sodium-glucose co-transporter 2 inhibition with empagliflozin improves cardiac function in non-diabetic rats with left ventricular dysfunction after myocardial infarction. Eur J Heart Fail.

[29] Lee HC, Shiou YL, Jhuo SJ, Chang CY, Liu PL, Jhuang WJ, Dai ZK, Chen WY, Chen YF, Lee AS (2019). he sodium-glucose co-transporter 2 inhibitor empagliflozin attenuates cardiac fibrosis and improves ventricular hemodynamics in hypertensive heart failure rats. Cardiovasc Diabetol.

[30] Adingupu DD, Göpel SO, Grönros J, Behrendt M, Sotak M, Miliotis T, Dahlqvist U, Gan LM, Jönsson-Rylander AC (2019). SGLT2 inhibition with empagliflozin improves coronary microvascular function and cardiac contractility in prediabetic ob/ob^−/−^ mice. Cardiovasc Diabetol.

[31] Li C, Zhang J, Xue M, Li X, Han F, Liu X, Xu L, Lu Y, Cheng Y, Li T, Yu X, Sun B, Chen L (2019). Empa attenuates ox stress and fibrosis in diabetic mice. Cardiovasc Diabetol.

[32] Wood IS, Trayhurn P (2003). Glucose transporters (GLUT and SGLT): expanded families of sugar transport proteins. Br J Nutr.

[33] Yoshii A, Nagoshi T, Kashiwagi Y, Kimura H, Tanaka Y, Oi Y, Ito K, Yoshino T, Tanaka TD, Yoshimura M (2019). Cardiac ischemia-reperfusion injury under insulin-resistant conditions: sGLT1 but not SGLT2 plays a compensatory protective role in diet-induced obesity. Cardiovasc Diabetol.

[34] Wang W, Liu N, Xin L, Ruan Y, Du X, Bai R, Dong J, Ma C (2019). Inhibition of miR-296-5p protects the heart from cardiac hypertrophy by targeting CACNG6. Gene Ther.

[35] Fang L, Ellims AH, Moore XL, White DA, Taylor AJ, Chin-Dusting J, Dart AM (2015). Circulating microRNAs as biomarkers for diffuse myocardial fibrosis in patients with hypertrophic cardiomyopathy. J Transl Med.

[36] Liu X, Wang Y, Zhang X, Zhang X, Guo J, Zhou J, Chai Y, Ma ZL (2019). MicroRNA-296-5p promotes healing of diabetic wound by targeting sodium-glucose transporter 2 (SGLT2). Diabetes Metab Res Rev.

[37] Li H, Gu Y, Zhang Y, Lucas MJ, Wang Y (2004). High glucose levels down-regulate glucose transporter expression that correlates with increased oxidative stress in placental trophoblast cells in vitro. J Soc Gynecol Investig.

[38] Oh CM, Cho S, Jang JY, Kim H, Chun S, Choi M, Park S, Ko YG (2019). Cardioprotective potential of an SGLT2 inhibitor against doxorubicin-induced heart failure. Korean Circ J.

[39] Ng KM, Lau YM, Dhandhania V, Cai ZJ, Lee YK, Lai WH, Tse HF, Siu CW (2018). Empagliflozin ammeliorates high glucose induced-cardiac dysfuntion in human iPSC-derived cardiomyocytes. Sci Rep.

[40] von Lewinski D, Gasser R, Rainer PP, Huber MS, Wilhelm B, Roessl U, Haas T, Wasler A, Grimm M, Bisping E, Pieske B (2010). Functional effects of glucose transporters in human ventricular myocardium. Eur J Heart Fail.

[41] Despa S, Islam MA, Weber CR, Pogwizd SM, Bers DM (2002). Intracellular Na(+) concentration is elevated in heart failure but Na/K pump function is unchanged. Circulation.

[42] Baartscheer A, Schumacher CA, Wüst RC, Fiolet JW, Stienen GJ, Coronel R, Zuurbier CJ (2017). Empagliflozin decreases myocardial cytoplasmic Na+ through inhibition of the cardiac Na+/H+ exchanger in rats and rabbits. Diabetologia.

[43] Kovacs CS, Seshiah V, Swallow R, Jones R, Rattunde H, Woerle HJ, Broedl UC, EMPA-REG PIO trial investigators (2014). Empagliflozin improves glycaemic and weight control as add-on therapy to pioglitazone or pioglitazone plus metformin in patients with type 2 diabetes: a 24-week, randomized, placebo-controlled trial. Diabetes Obes Metab.

[44] van Dalen EC, Caron HN, Dickinson HO, Kremer LC. Cardioprotective interventions for cancer patients receiving anthracyclines. Cochrane Database Syst Rev. 2011; (6):CD003917. 10.1002/14651858.cd003917.pub4. 10.1002/14651858.CD003917.pub4 PMC6457676 21678342

[45] Vejpongsa P, Yeh ET (2014). Prevention of anthracycline-induced cardiotoxicity: challenges and opportunities. J Am Coll Cardiol.

[46] Kalam K, Marwick TH (2013). Role of cardioprotective therapy for prevention of cardiotoxicity with chemotherapy: a systematic review and meta-analysis. Eur J Cancer.

[47] Akpek M, Ozdogru I, Sahin O, Inanc M, Dogan A, Yazici C, Berk V, Karaca H, Kalay N, Oguzhan A, Ergin A (2015). Protective effects of spironolactone against anthracycline-induced cardiomyopathy. Eur J Heart Fail.

[48] Maurea N, Coppola C, Ragone G, Frasci G, Bonelli A, Romano C, Iaffaioli RV (2010). Women survive breast cancer but fall victim to heart failure: the shadows and lights of targeted therapy. J Cardiovasc Med.

[49] Gulati G, Heck SL, Røsjø H, Ree AH, Hoffmann P, Hagve TA, Norseth J, Gravdehaug B, Steine K, Geisler J, Omland T (2017). Neurohormonal blockade and circulating cardiovascular biomarkers during anthracycline therapy in breast cancer patients: results from the PRADA (prevention of cardiac dysfunction during adjuvant breast cancer therapy) study. J Am Heart Assoc.

[50] Boekhout AH, Gietema JA, Milojkovic Kerklaan B, van Werkhoven ED, Altena R, Honkoop A, Los M, Smit WM, Nieboer P, Smorenburg CH, Mandigers CM, van der Wouw AJ, Kessels L, van der Velden AW, Ottevanger PB, Smilde T, de Boer J, van Veldhuisen DJ, Kema IP, de Vries EG, Schellens JH (2016). Angiotensin II-receptor inhibition with candesartan to prevent trastuzumab-related cardiotoxic effects in patients with early breast cancer: a randomized clinical trial. JAMA Oncol.

[51] Pituskin E, Haykowsky M, Mackey JR, Thompson RB, Ezekowitz J, Koshman S, Oudit G, Chow K, Pagano JJ, Paterson I (2011). Rationale and design of the Multidisciplinary Approach to Novel Therapies in Cardiology Oncology Research Trial (MANTICORE 101–Breast): a randomized, placebo-controlled trial to determine if conventional heart failure pharmacotherapy can prevent trastuzumab-mediated left ventricular remodeling among patients with HER2+ early breast cancer using cardiac MRI. BMC Cancer.

