# Supplementary material for: Empagliflozin prevents doxorubicin-induced myocardial dysfunction
Source: Cardiovasc Diabetol. 2020 May 15;19:66. doi: 10.1186/s12933-020-01040-5 (PMC7229599; doi:10.1186/s12933-020-01040-5)
Supplement: Supplementary file 1 — Additional file 1.Extended methods. Extended version of study methodology, including all details that the limited word count available did not allow to keep in the main manuscript. Table S1. MicroRNAs targeting SGLT2. List of microRNAs potentially targeting SGLT2 within the highest context score percentiles. The upper row shows the microRNA targeting a conserved site of SGLT2, while the remaining microRNAs target poorly conserved sites. Figure S1. Study timeline. The figure depicts the study timeline with the breakdown of mice into three groups. Figure S2. Systemic blood pressure. At the sixth week, both systolic (panel A) and diastolic blood pressure (panel B) was significantly lower in the DOX group compared to DOX+EMPA. Figure S3. Histological examples of doxorubicin myocardial injuries. The figure shows the characteristic attenuation of fibrillar bands observed after treatment with doxorubicin, in some morphological variants (lower left and mid panels and upper mid panel). Masson’s trichrome staining highlights the degree of fibrosis in hearts from control group (upper right panel) and hearts from the DOX group (lower right panel). Figure S4. Expression of SGLT-1 in mice hearts. Results RT-PCR showing the expression levels of SGLT-1 in different mouse hearts. Figure S5. Expression of SGLT-2 in mice hearts. Immunohistochemistry shows the expression of SGLT-2 in mice hearts. SGLT-2 expression showed a 75% reduction in DOX mice (upper right panel), compared to baseline. This effect was significantly attenuated in EMPA+DOX mice (lower right panel). [file 12933_2020_1040_MOESM1_ESM.docx]

**Additional Files**

**EXTENDED METHODS**

***Animal model.*** Male 8 weeks-old C57Bl/6 mice were randomly assigned to one of the following treatments: saline plus DOX (DOX group; n=14), or EMPA plus DOX (DOX+EMPA group; n=14), or furosemide (20 mg/kg daily by oral gavage for 5 weeks) plus DOX (DOX+FURO group; n=7). DOX was injected intraperitoneally at weekly doses of 5 mg/kg for five weeks. EMPA (10 mg/kg) was administered daily by oral gavage for 5 weeks. In addition, a further control group of animals (n=7) received no treatment at all (Supplementary Figure 1). Our animal protocol was designed to minimize pain and discomfort, in compliance with requests by the local Animal Wellness and Welfare Committee (O.P.B.A.).

All mice were monitored for body weight and heart function under basal conditions and 6 weeks later. They were housed in standard plexiglass cages and maintained on a 12 h light/12 h dark cycle in a temperature-controlled room (22±2˚C), with access to food and water ad libitum. At the end of the experiment, animals were euthanized, and hearts processed for histological evaluations. All animal experiments were performed according to European Directive 63/2010/UE and Italian Law (DL 26/2014, authorized by the Minister of Health, Italy).

Chronic myocardial injury was obtained using a standardized model of DOX-induced toxicity, already described in previous studies (15). According to the protocol, mice were administered with weekly injections of Dox (5 mg/kg) for 5 weeks (Supplementary Figure 1).

***Urine volume and glycosuria measurements by metabolic cages.*** For evaluating urine volume and glycosuria, additional 10 conscious mice were individually placed in metabolic cages in a soundproof room at 25 degrees temperature with a 12/12 hr light-dark cycle. They were randomized in two groups: one group was treated with empagliflozin (n=6), and the other with placebo (n=4). Each mouse was provided with free access to food and water. After mice were acclimatized for 3 days in the cages, the subsequent 48 hr data of voided urine and water intake were continuously recorded into a computer and analysed. Evaluated parameters included water intake (μl/day), urine volume (μl/day), and glycosuria (μg/day).

***Standard echocardiographic analyses.*** *In vivo,* cardiac function was assessed by transthoracic echocardiography in sedated mice by using a Vevo 2100 (Visualsonics, Toronto, Canada) high-resolution imaging system, with 22-55 hMHz transducer (9). Mice were sedated and placed in supine position on a temperature-controlled surgical table to maintain rectal temperature at 37°C. A light anesthesia protocol (1% isoflurane) was adopted to minimize anesthesia-induced depression of LV function, as previously described by others (16-17). Continuous electrocardiographic monitoring was obtained via limb electrodes; limbs were kept in position using a small amount of silk plaster.

Cardiac function was evaluated by echocardiography under basal conditions and at the end of the protocol 6 weeks later. LV echocardiography was assessed in both parasternal long-axis and short-axis views at a frame rate of 233 Hz. : LVIDd and LVIDs, and then FS and EF, were measured from the LV M-mode at the mid papillary muscle level order to standardize measurement protocol and to ensure the measurements are taken at the same LV level. This approach is widespread as it has been reported to maximize reproducibility, allowing to measure septum at the same site in all exams (17). Measurements of diastolic LV internal dimensions (LVIDd) and systolic LV internal dimensions (LVIDs) were averaged from three to five beats. LVIDd and LVIDs were measured from the LV M-mode at the mid papillary muscle level.

Fractional shortening (FS) percentage was calculated as [(LVIDd−LVIDs)/LVIDd]×100, and ejection fraction (EF) percentage was calculated as [(EDvol−ESvol)/EDvol]×100.

***Speckle Tracking Analysis.*** Acquired B-mode loops in both parasternal long-axis and short-axis views were imported into VevoStrain software (VisualSonics, Toronto, ON, Canada). Three consecutive cardiac cycles were selected and adequate tracing of the endocardium was performed (9). Strain was evaluated in three axes: radial, circumferential and longitudinal.

***Morphological examination and cardiac fibrosis analysis.*** Hearts from the studied mice were fixed with 10% formalin and embedded in paraffin. Sections were stained with hematoxylin and eosin (H&E) for morphological tissue analysis. Interstitial fibrosis was evaluated by means of the Masson’s Trichrome staining. The fibrotic area was measured using a computer-assisted image analysis system (Nikon NIS ELEMENTS BRV, Melville, NY, USA) and expressed as a percentage of total area. The percentage of red staining was calculated from all samples, with two sections for each sample and five images for each section.

***Blood pressure measurements.*** Non-invasive blood pressure measurements were performed in the three study groups at baseline and at the end of the treatment, by means of BP-2000 Blood Pressure Analysis System.

***Blood samples analysis.*** Blood samples were obtained from the retro-orbital vein and analysed using the point-of-care analyzer (Samsung LABGEO PT10 (Thermo Fischer Scientific, Henningsdorf, Germany). Levels of Troponin, BNP and glucose were analysed under basal conditions and 6 weeks later.

***RNA Extraction and Quantitative Real-Time Polymerase Chain Reaction.*** After RNA extraction from LV tissue and cDNA synthesis, quantitative real-time PCR was performed with SYBR-greenbased detection of double-stranded DNA using mouse specific primers and the ABI-7500 real-time PCR system (Applied Biosystems) (20). For each set of primers, a no template control and a no reverse transcription control was included. Post-amplification dissociation curves were performed to verify the presence of a single amplification product in the absence of genomic DNA contamination. Results were expressed as fold change in mRNA expression compared to the control (reference group) using reference genes.

***Protein Extraction and Western Blot.*** Proteins were prepared from mouse LV tissue using a lysis buffer containing protease inhibitors and phosphatase inhibitor cocktails. Cell lysates were separated by SDS-PAGE and transferred onto nitrocellulose membrane. The membranes were blocked with 5% non-fat milk and incubated overnight at 4 °C with the following primary antibodies: SGLT-1, SGLT-2, ERK, phospho-ERK. The membranes were incubated with appropriate secondary antibodies and signal intensities will be visualized by enhanced chemiluminescence. Films from at least three independent experiments were scanned and densities of the immunoreactive bands were evaluated using the NIH Image software. GAPDH and β-actin were used as internal controls.

***Statistical Analysis.*** Continuous data were expressed as mean ± SD. For paired comparisons of continuous variables the nonparametric Wilcoxon test was used (21). For comparisons between study groups the nonparametric Mann-Whitney and Kruskal-Wallis tests were applied (21-22). Repeated measures ANOVA was used for all baseline to end-of-study comparisons. A p value below 0.05 was considered statistically significant, with Bonferroni correction for multiple comparisons between the groups.

**ADDITIONAL FIGURE LEGENDS**

**Figure S1. Study timeline.**

The figure depicts the study timeline with the breakdown of mice into three groups.

**Figure S2. Systemic blood pressure.**

At the sixth week, both systolic (panel A) and diastolic blood pressure (panel B) was significantly lower in the DOX group compared to DOX+EMPA.

**Figure S3. Histological examples of doxorubicin myocardial injuries.**

The figure shows the characteristic attenuation of fibrillar bands observed after treatment with doxorubicin, in some morphological variants (lower left and mid panels and upper mid panel). Masson’s trichrome staining highlights the degree of fibrosis in hearts from control group (upper right panel) and hearts from the DOX group (lower right panel).

**Figure S4. Expression of SGLT-1 in mice hearts.**

Results RT-PCR showing the expression levels of SGLT-1 in different mouse hearts.

**Figure S5. Expression of SGLT-2 in mice hearts.**

Immunohistochemistry shows the expression of SGLT-2 in mice hearts. SGLT-2 expression showed a 75% reduction in DOX mice (upper right panel), compared to baseline. This effect was significantly attenuated in EMPA+DOX mice (lower right panel).

**Table S1. MicroRNAs targeting SGLT2**

| **miRNA** | **seed match** | **context score percentile** |
| --- | --- | --- |
| **Conserved sites** |  |  |
| hsa-miR-296-5p | 8mer | 99 |
| **Poorly conserved sites** | |  |
| hsa-miR-584-5p | 7mer-1A | 75 |
| hsa-miR-4474-3p | 7mer-m8 | 90 |
| hsa-miR-7974 | 7mer-m8 | 67 |
| hsa-miR-485-5p | 8mer | 97 |
| hsa-miR-6884-5p | 8mer | 97 |
| hsa-miR-3975 | 7mer-1A | 83 |
| hsa-miR-3188 | 7mer-1A | 74 |
| hsa-miR-2467-5p | 7mer-1A | 69 |
| hsa-miR-4316 | 8mer | 98 |
| hsa-miR-1294 | 7mer-1A | 64 |
| hsa-miR-4677-3p | 7mer-m8 | 58 |
| hsa-miR-1914-5p | 7mer-1A | 74 |
| hsa-miR-7152-5p | 7mer-m8 | 53 |
| hsa-miR-4286 | 7mer-m8 | 90 |
| hsa-miR-6802-3p | 7mer-m8 | 91 |
| hsa-miR-6784-3p | 7mer-m8 | 90 |
| hsa-miR-6862-3p | 7mer-m8 | 89 |
| hsa-miR-4268 | 7mer-m8 | 87 |
| hsa-miR-484 | 7mer-m8 | 96 |
| hsa-miR-3155a | 7mer-m8 | 92 |
| hsa-miR-3155b | 7mer-m8 | 92 |
| hsa-miR-6886-5p | 7mer-m8 | 89 |
| hsa-miR-3663-3p | 7mer-m8 | 85 |
| hsa-miR-1343-5p | 8mer | 98 |
| hsa-miR-939-5p | 8mer | 98 |
| hsa-miR-3175 | 7mer-1A | 89 |
| hsa-miR-3150a-3p | 7mer-m8 | 77 |
| hsa-miR-6763-5p | 7mer-m8 | 75 |
| hsa-miR-6825-5p | 7mer-1A | 45 |
| hsa-miR-8089 | 8mer | 99 |
| hsa-miR-4700-5p | 8mer | 99 |
| hsa-miR-4667-5p | 8mer | 99 |
| hsa-miR-637 | 7mer-1A | 88 |
| hsa-miR-7155-5p | 7mer-1A | 86 |
| hsa-miR-4314 | 7mer-1A | 76 |
| hsa-miR-627-3p | 7mer-m8 | 38 |
| hsa-miR-6832-3p | 7mer-m8 | 37 |
| hsa-miR-6756-3p | 7mer-1A | 62 |
| hsa-miR-3127-3p | 7mer-1A | 59 |
| hsa-miR-6826-3p | 7mer-1A | 76 |
| hsa-miR-6734-3p | 7mer-m8 | 85 |
| hsa-miR-6756-3p | 7mer-m8 | 81 |
| hsa-miR-3127-3p | 7mer-m8 | 79 |
| hsa-miR-210-5p | 7mer-1A | 81 |
| hsa-miR-4749-3p | 7mer-1A | 77 |
| hsa-miR-1913 | 7mer-m8 | 92 |
| hsa-miR-324-3p | 7mer-m8 | 91 |
| hsa-miR-6890-3p | 7mer-m8 | 88 |
| hsa-miR-1273g-3p | 7mer-m8 | 59 |
| hsa-miR-4286 | 7mer-m8 | 70 |
| hsa-miR-6802-3p | 7mer-m8 | 87 |
| hsa-miR-6784-3p | 8mer | 97 |
| hsa-miR-6862-3p | 8mer | 97 |
| hsa-miR-1229-3p | 8mer | 96 |
| hsa-miR-6511b-3p | 7mer-1A | 84 |
| hsa-miR-6511a-3p | 7mer-1A | 82 |
| hsa-miR-659-5p | 7mer-m8 | 85 |
| hsa-miR-505-5p | 7mer-m8 | 77 |
| hsa-miR-6827-5p | 7mer-m8 | 93 |
| hsa-miR-1343-5p | 7mer-m8 | 95 |
| hsa-miR-939-5p | 7mer-m8 | 95 |
| hsa-miR-3179 | 7mer-m8 | 87 |
| hsa-miR-3154 | 7mer-m8 | 81 |
| hsa-miR-6770-5p | 7mer-m8 | 75 |
| hsa-miR-5703 | 7mer-m8 | 75 |
| hsa-miR-4434 | 7mer-m8 | 70 |
| hsa-miR-4516 | 7mer-m8 | 67 |
| hsa-miR-6878-5p | 7mer-m8 | 26 |
| hsa-miR-6746-5p | 7mer-m8 | 89 |
| hsa-miR-4493 | 7mer-m8 | 85 |
| hsa-miR-4476 | 7mer-m8 | 84 |
| hsa-miR-6876-5p | 7mer-m8 | 80 |
| hsa-miR-3125 | 7mer-m8 | 71 |
| hsa-miR-6859-5p | 7mer-m8 | 40 |
| hsa-miR-3916 | 7mer-m8 | 35 |
| hsa-miR-6847-5p | 7mer-m8 | 85 |
| hsa-miR-2467-3p | 7mer-m8 | 86 |
| hsa-miR-6511a-5p | 7mer-m8 | 80 |
| hsa-miR-1910-3p | 7mer-m8 | 49 |
| hsa-miR-4692 | 7mer-m8 | 82 |
| hsa-miR-4514 | 7mer-m8 | 82 |
| hsa-miR-4673 | 7mer-m8 | 82 |
| hsa-miR-4645-5p | 7mer-m8 | 65 |
| hsa-miR-874-5p | 7mer-m8 | 76 |
| hsa-miR-6724-5p | 7mer-m8 | 84 |
| hsa-miR-6773-5p | 7mer-m8 | 83 |
| hsa-miR-194-3p | 7mer-m8 | 63 |
| hsa-miR-6499-3p | 7mer-1A | 77 |
| hsa-miR-1245b-3p | 7mer-1A | 92 |
| hsa-miR-192-3p | 7mer-m8 | 84 |
| hsa-miR-5002-3p | 8mer | 99 |
| hsa-miR-134-5p | 7mer-m8 | 92 |
| hsa-miR-3118 | 7mer-m8 | 89 |
| hsa-miR-4747-5p | 7mer-1A | 83 |
| hsa-miR-5196-5p | 7mer-1A | 80 |
| hsa-miR-4755-3p | 7mer-m8 | 95 |
| hsa-miR-4726-5p | 8mer | 95 |
| hsa-miR-4640-5p | 8mer | 95 |
| hsa-miR-6508-3p | 7mer-1A | 78 |
| hsa-miR-654-5p | 7mer-m8 | 90 |
| hsa-miR-541-3p | 7mer-m8 | 89 |
| hsa-miR-6766-5p | 7mer-m8 | 65 |
| hsa-miR-6756-5p | 7mer-m8 | 64 |
| hsa-miR-3656 | 7mer-m8 | 89 |
| hsa-miR-1184 | 7mer-m8 | 95 |
| hsa-miR-509-5p | 7mer-m8 | 95 |
| hsa-miR-509-3-5p | 7mer-m8 | 94 |
| hsa-miR-4418 | 7mer-m8 | 91 |
| hsa-miR-4761-3p | 7mer-1A | 95 |
| hsa-miR-7843-5p | 7mer-1A | 93 |
| hsa-miR-4632-5p | 7mer-1A | 93 |
| hsa-miR-6879-5p | 7mer-1A | 93 |
| hsa-miR-6735-5p | 7mer-1A | 90 |
| hsa-miR-4436b-3p | 7mer-1A | 90 |
| hsa-miR-2909 | 7mer-m8 | 93 |
| hsa-miR-1296-5p | 7mer-1A | 77 |
| hsa-miR-3714 | 7mer-m8 | 97 |
| hsa-miR-548an | 7mer-m8 | 92 |
| hsa-miR-6733-5p | 7mer-m8 | 94 |
| hsa-miR-6739-5p | 7mer-m8 | 93 |
| hsa-miR-3153 | 7mer-m8 | 92 |
| hsa-miR-6783-5p | 7mer-m8 | 96 |

**Figure S1**


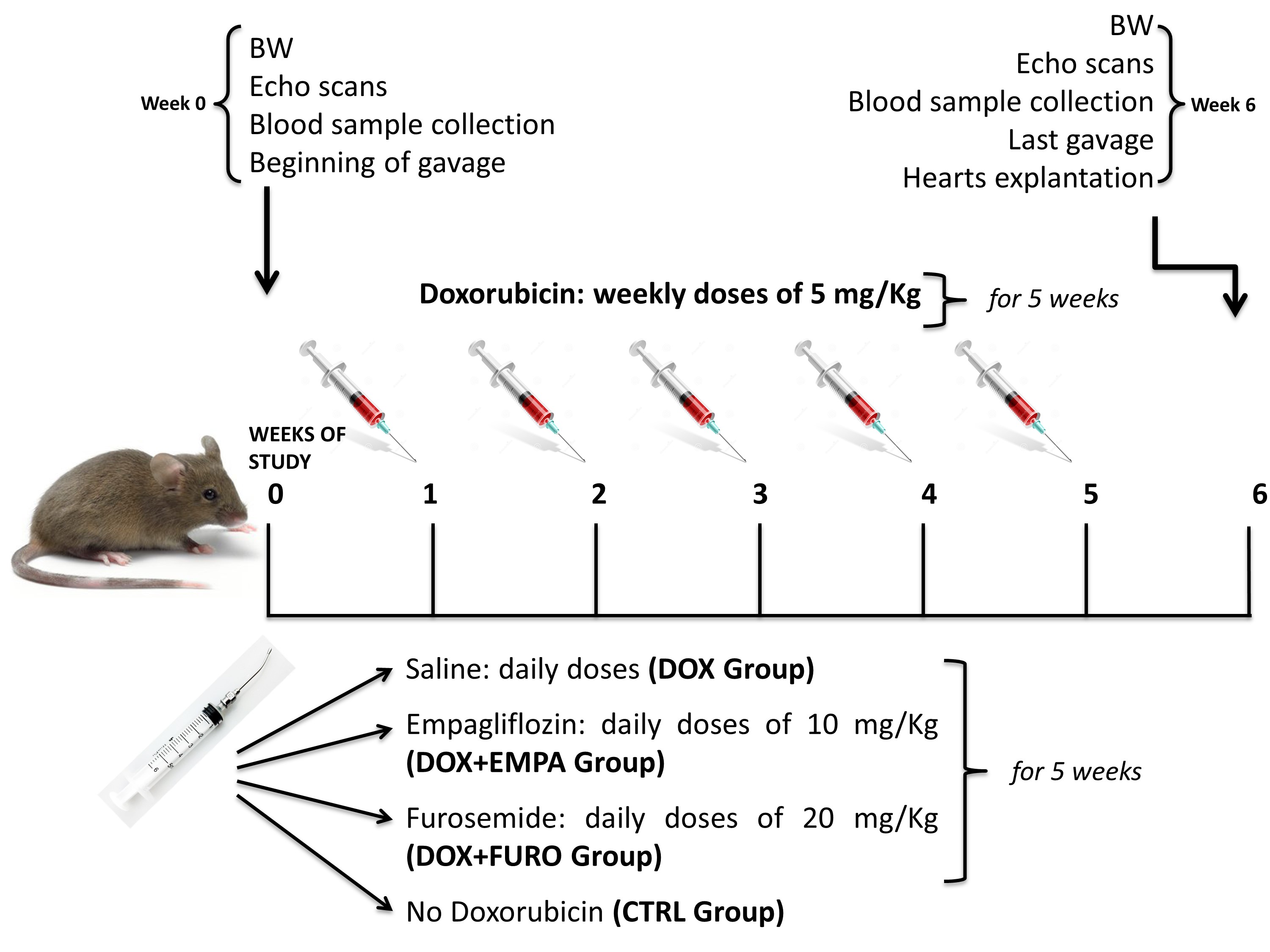


**Figure S2**


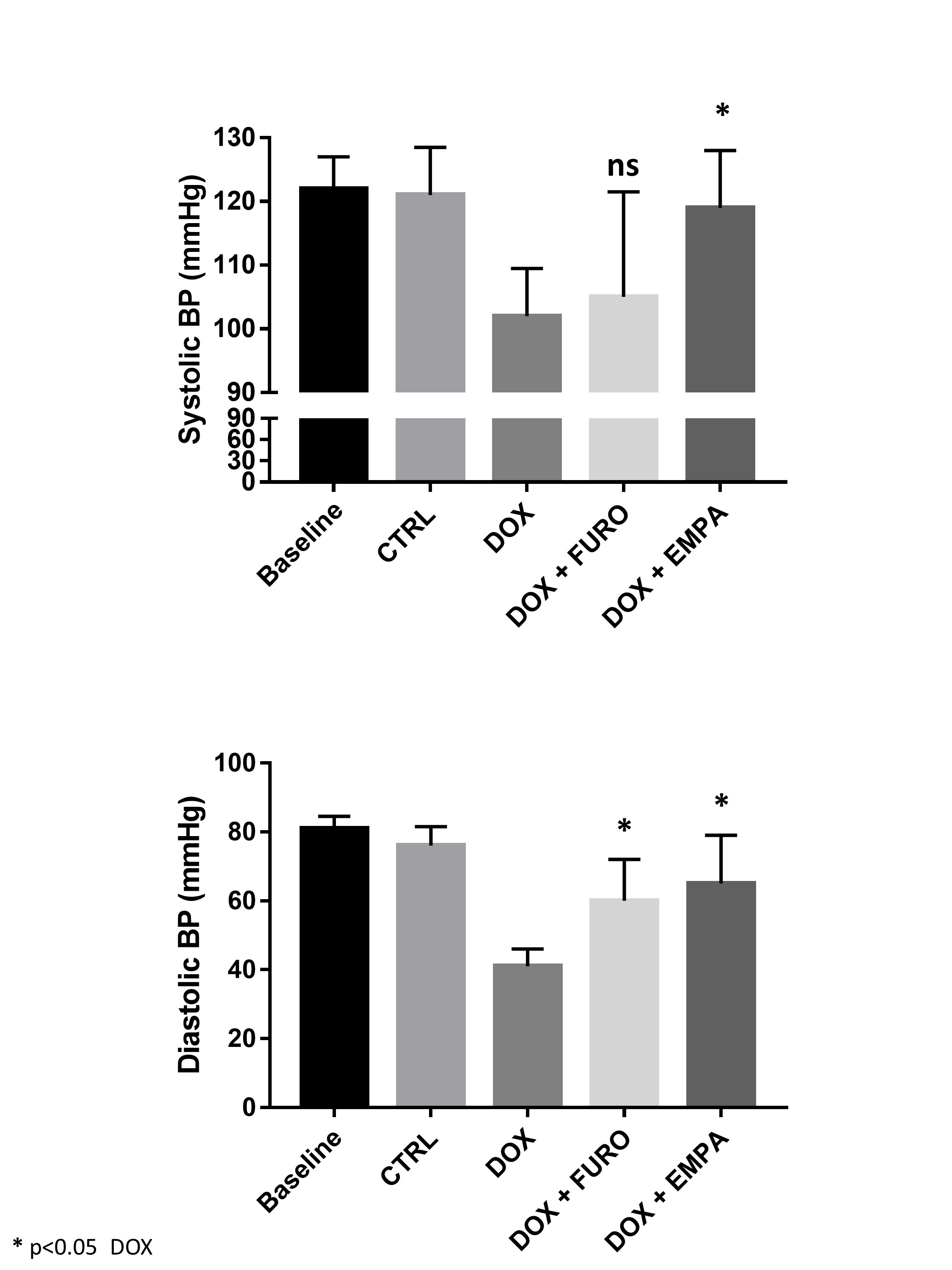


**Figure S3**


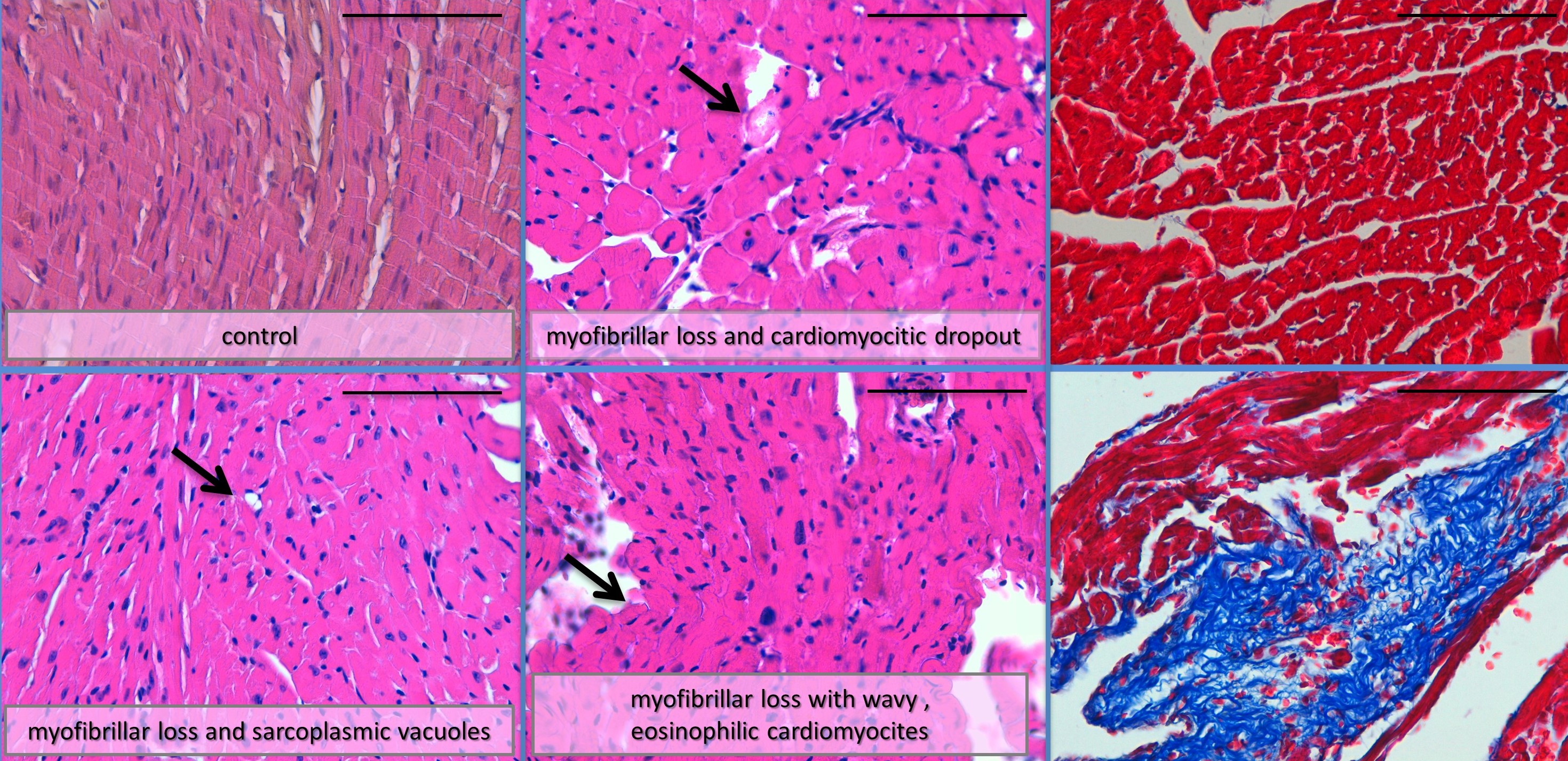


**Figure S4**


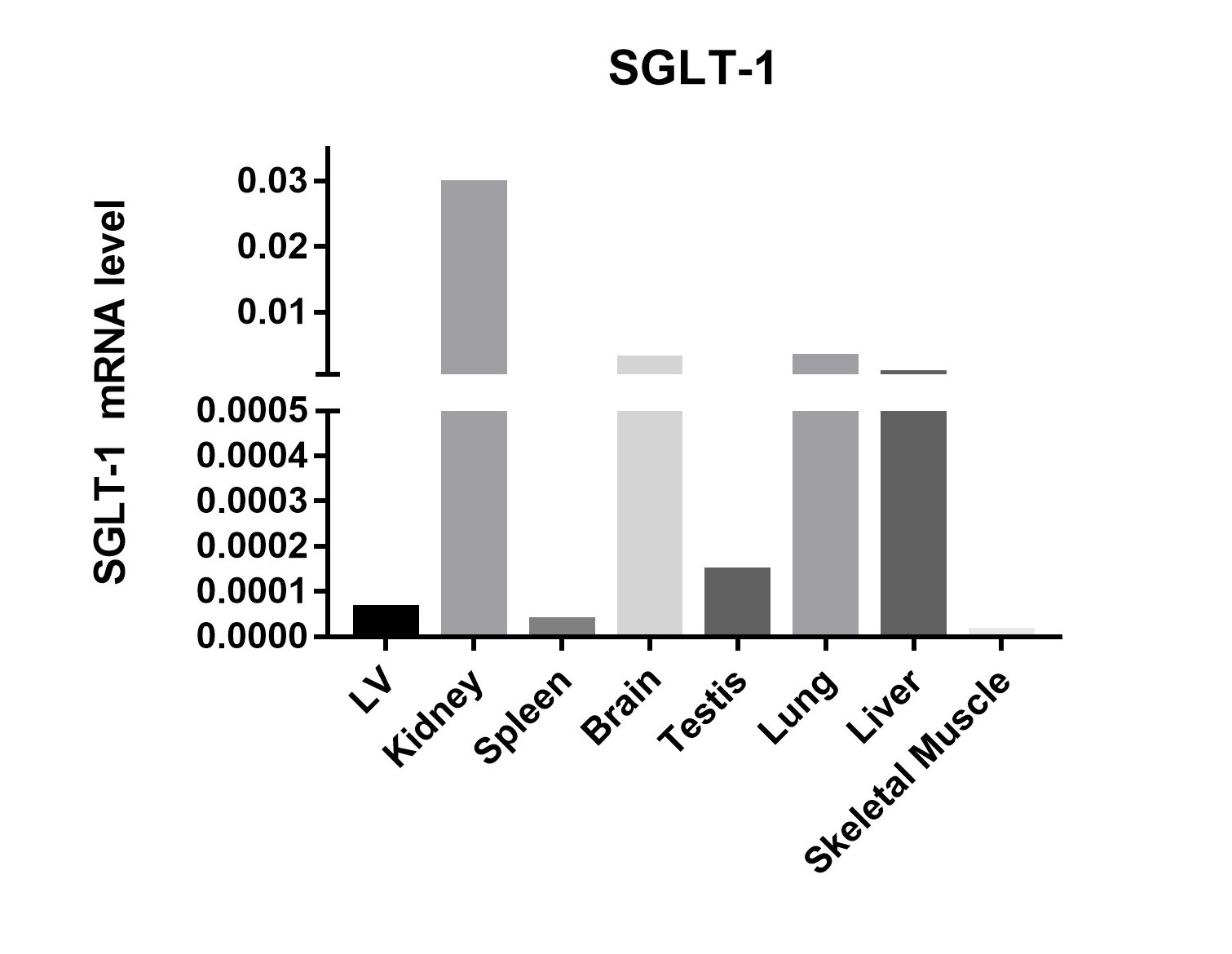


**Figure S5
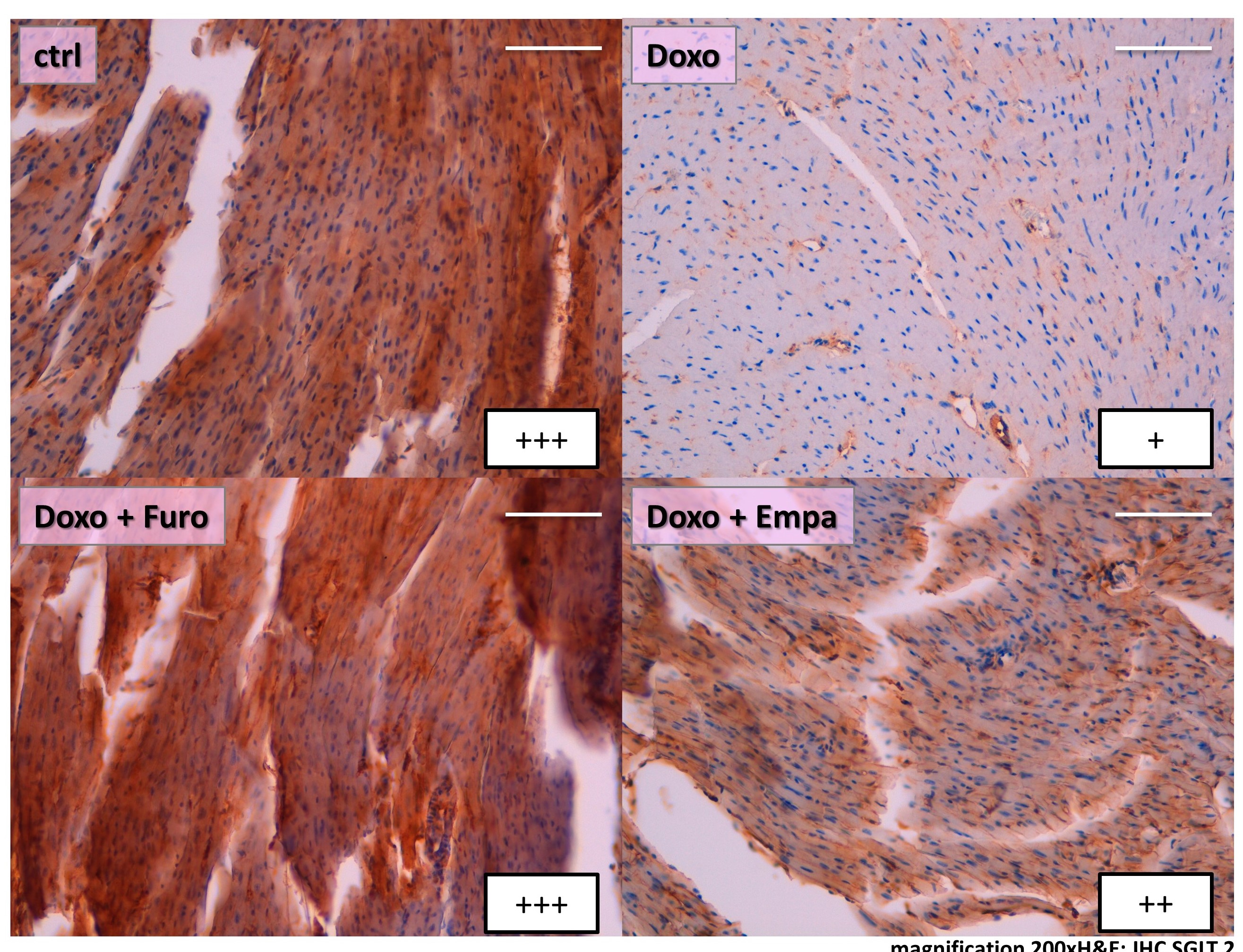
**
